# Vaping-Induced Lung Injury: A Case of Lipoid Pneumonia Associated with E-Cigarettes Containing Cannabis

**DOI:** 10.1155/2020/7151834

**Published:** 2020-04-03

**Authors:** Bryno Gay, Zachary Field, Sachin Patel, Rodrigo Murillo Alvarez, Wael Nasser, Mario Madruga, S. J. Carlan

**Affiliations:** ^1^Department of Medicine, Orlando Regional Healthcare, Orlando, Florida, USA; ^2^Department of Pulmonary, Critical Care and Sleep Medicine, Orlando Regional Healthcare, Orlando, USA; ^3^Department of Pathology, Orlando Regional Healthcare, Orlando, USA; ^4^Division of Academic Affairs and Research, Orlando Regional Healthcare, Orlando, USA

## Abstract

Electronic cigarette, or vaping product use-associated lung injury (EVALI), is a group of lung disorders associated with vaping and e-cigarette products that has previously been categorized as a diagnosis of exclusion and best described as an exogenous lipoid pneumonia or chemical pneumonitis. Here, we describe the onset of an exogenous cause of lipoid pneumonia in an otherwise healthy patient using cannabis-containing electronic cigarettes. We explore similarities in the clinical case, define a common clinical presentation with progression of disease, characteristic radiographic findings along with pathological diagnosis and management.

## 1. Introduction

Electronic cigarettes were introduced into the United States market in 2007. Since then, both nicotine-containing electronic cigarettes and cannabis-containing electronic cigarettes have seen a rapid rise in popularity [1, 2]. Frequently marketed as a less harmful alternative to conventional smoking, many chronic tobacco and cannabis smokers as well as new users have contributed to the rise in demand. In this paper, we will use the term “vape pen” to mean an electronic cigarette containing cannabis and “e-cigarette” to mean an electronic cigarette containing nicotine. Vape pens operate in the same way as e-cigarettes. However, unlike its nicotine counterpart, tetrahydrocannabinol is not widely available as a purified chemical. Instead, vape pens contain extracts of cannabis plant material. Known variously as cannabis extract, cannabis concentrate, THC oil, hash oil, and butane-hash oil, these extracts are not diluted in propylene glycol or glycerol like nicotine due to their hydrophobic properties. Instead, various forms of oils including vegetable oils, terpenes, and tocopheryl acetate (vitamin E acetate) have been reported as diluents. In most reported cases of EVALI, additional flavoring additives are also added to products [3].

Although many of these diluent agents and flavorings have been “generally recognized as safe” for oral ingestion by the FDA, recent research shows that when heated to form an aerosol and inhaled, conditions including bronchitis, bronchiolitis, acute hypoxic respiratory distress, lipoid-associated pneumonia, and pneumonitis may result [1, 4, 5]. As a relatively new product, the safety profile and long-term effects of vape pens and e-cigarettes remain largely undefined.

The outbreak of severe and acute pulmonary disease associated with the use of cannabis extract-containing vape pens and nicotine e-cigarettes has grown to 2,711 reported cases according to the most up-to-date data retrieved from the Center for Disease Control and Prevention (CDC) website posted on January 20, 2020 [6]. In fact, all 50 states, the District of Columbia, and two U.S. territories (Puerto Rico and the U.S. Virgin Islands) have reported severe and acute pulmonary disease associated with e-cigarettes with 27 confirmed associated deaths [6].

Although a definitive cause is postulated to be a chemical exposure, more specifically from substances within the cannabis vape pens [5, 7], the acute incidence of pulmonary disease has brought national attention to this epidemic and warrants a continued investigation into these products, their contents, and medical management for EVALI, especially those purchased from informal sources (i.e., black market products) as reported in the case below.

### 1.1. Case Report

A 46-year-old male with no significant medical history presented with shortness of breath and flu-like symptoms including generalized weakness, fatigue, chills, diaphoresis, fever, and cough. The patient denied any recent sick contacts, travel history, history of shortness of breath, or history of lung disease. The patient reported only smoking conventional marijuana for 20 plus years, smoking 2 to 3 times per day, and had approximately a 4-6-month history of vape pen usage, with and without flavor additives. The cannabis vape pens were purchased in bulk from an informal source. The patient reported a less than 1-year history of conventional cigarette use and quit over 20 years ago. He denied the use of chewing tobacco, electronic cigarettes containing nicotine, or any other nicotine-containing products during this time.

On arrival, the patient was afebrile with a respiratory rate of 20 breaths per minute, oxygen saturation was 96% on ambient air, blood pressure was 126/82 mmHg, and heart rate was 96 beats per minute. Over the next 4 hours, the patient rapidly deteriorated into acute hypoxic respiratory failure with hypoxia down to 86% while on 2 liters nasal cannula. The patient was increased to 13 liters high-flow nasal cannula, started on empiric antibiotics, and intravenous methylprednisolone 125 mg which corrected the hypoxia with sustained oxygen saturation of 94%.

**Figure 1:**
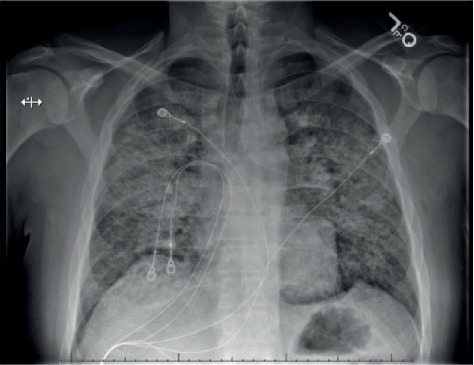
Chest X-ray showing extensive patchy bilateral consolidation.

**Figure 2:**
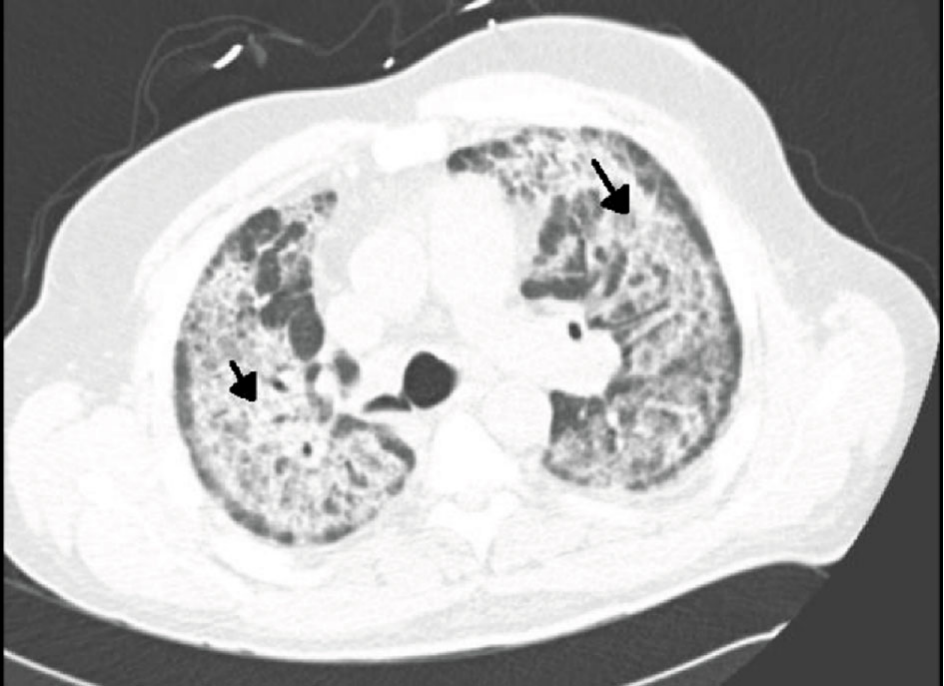
CT of the thorax showing extensive bilateral infiltrates and ground glass type densities (black arrow) sparing the periphery.

Physical exam was remarkable for diaphoresis, tachypnea, labored breathing with accessory muscle use, and diffuse inspiratory crackles. An arterial blood gas showed a pH of 7.51 [7.35-7.45], pCO2 38 [35-45 mmHg], pO2 84 [80-100 mmHg], and HCO_3_ of 30.3 mmol/L [22-26 mmol/L]. Lab work showed a venous lactic acidosis at 2.3 mmol/L [0.9-1.7 mmol/L], white blood cell count of 14.8 × 10^3^/*μl* [4.4 − 10.5 × 10^3^/*μl*], and a normal sedimentation rate. Chest X-ray noted diffuse, bilateral pulmonary consolidation sparing the apices, and bases with bronchopulmonary cuffing (Figure 1). Computed tomography (CT) imaging showed extensive bilateral airspace disease with increased reticulation, traction bronchiectasis, and ground glass opacities but noted sparing of apical spaces bilaterally (Figure 2).

**Figure 3:**
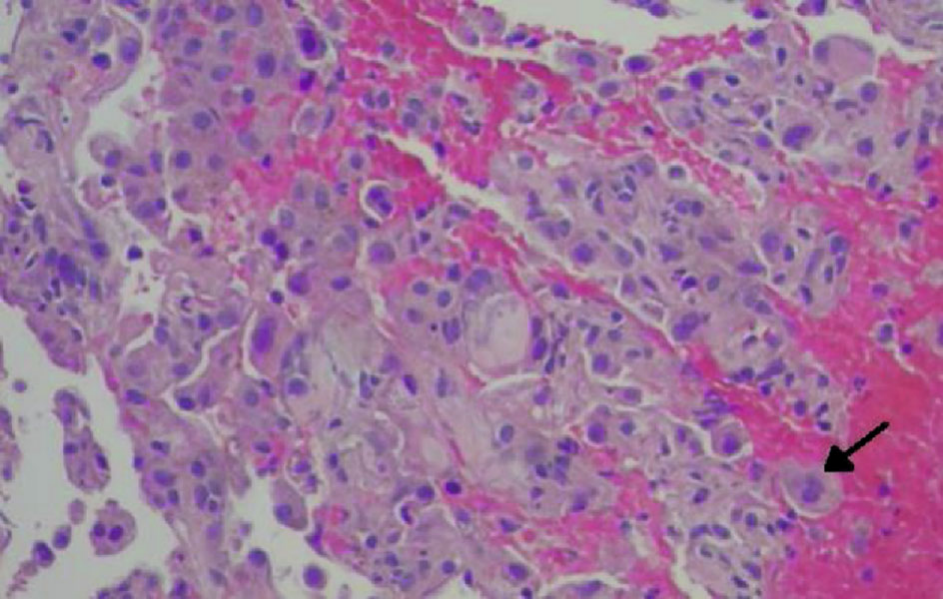
Lung biopsy showing organizing pneumonia and foamy macrophages (black arrow). (Hematoxylin-eosin, original magnification ×400).

**Figure 4:**
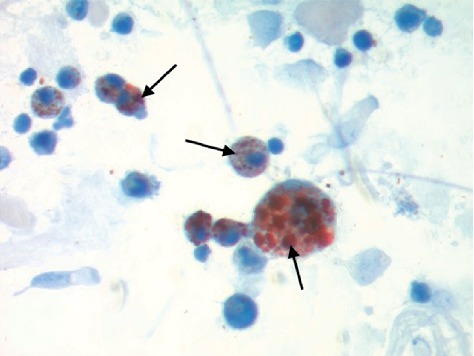
Bronchoalveolar lavage fluid stained with Oil red O highlights numerous macrophages containing lipid deposits, marked with black arrows (lipid-laden macrophages).

Further lab work including blood and sputum cultures, HIV, urine Histoplasma, Legionella, Pneumococcal antigens, and respiratory viral panel were all negative. Serologies—coccidiomycosis, histoplasmosis, and pneumocystis—were also negative. Bronchoscopy with transbronchial biopsies were performed and a sample from the right lower lobe was significant for intra-alveolar inflammation including foamy macrophages and activated type II pneumocytes (Figure 3). These findings were consistent with organizing pneumonia. A histopathology examination on bronchoalveolar lavage (BAL) sample stained with Oil red O showed numerous lipid-laden macrophages and intra-alveolar macrophages containing blackish-brown pigment (Figure 4). This was consistent with a diagnosis of lipoid pneumonia. Additional testing was negative for AFB stain, cytomegalovirus, herpes simplex antigen, and aspergillus antigen. During the hospital course, the patient improved over a six-day regimen of bronchodilators, steroids, and empiric antibiotics with supportive care of oxygen supplementation. He was discharged on the same regimen for 14 days with follow-up with pulmonology and repeat CT imaging in six weeks.

## 2. Discussion

Lipoid pneumonia is an unusual disease caused by the presence of lipids in the alveoli and is a chronic foreign body reaction to fat. It can be classified into two groups depending on an exogenous versus endogenous source of lipid or oil and host tissue reactions to the inhaled substances according to their chemical characteristics. The clinical presentation is unpredictable with progressive, subtle respiratory symptoms ranging from dyspnea and cough to severe life-threatening diseases [8–11].

Consequently, making a diagnosis of exogenous lipoid pneumonia requires a high degree of clinical suspicion. Radiographic findings consistent with EVALl include a crazy-paving pattern, interlobular septal thickening, diffuse infiltrates with a range of “ground glass” opacities, and nodular or “tree-in-bud” apical surfaces [12–14]. The presence of lipid-laden macrophages in the sputum or BAL specimen are also consistent with diagnosis of EVALI; however, high lipid-laden macrophages and high lipid-laden alveolar macrophage index (LLAMI) are nonspecific findings and can be found in various lung diseases [15]. Therefore, the addition of foamy macrophages, extracellular oily droplets, and macrophage with large cytoplasmic vacuoles contrasting to small vacuoles in the endogenous forms can guide a more specific diagnosis of exogenous lipoid pneumonia [16] which may also contain inflammatory cells similar to a foreign body reaction with a proliferative fibrosis and black pigmented dye in macrophages [17]. Lastly, vacuolated macrophages will stain orange with Sudan stain or red with Oil red O stain [15] as seen in BAL sample collected in this case (Figure 4).

THC-containing vape pens have become increasingly popular among adolescents. Current data from the Center of Disease Control (CDC) reports 27 deaths associated with EVALI. Interestingly, demographic data from a sample of 2,711 patients illustrated a majority of male patients (66%). Of the total reported cases 15% were below 18 years old, 37% were between 18 and 24 years old, 24% were between 25 and 34 years old, and 24% were 35 years old or older [6]. Approximately 82% reported using THC-containing products, 33% reported exclusive use of THC-containing products, 57% reported using nicotine-containing products, and 14% reported exclusive use of nicotine-containing products [6]. Of the EVALI patients who reported using THC-containing products, 78% reported acquiring products from informal sources while 69% of EVALI patients who reported using nicotine-containing products acquired theirs from an established business [6].

THC vape pens are a prefilled cartridge of cannabis extract, which may include diluents and flavorings, with a battery-operated heating system [7]. Although classified as “generally recognized as safe” by the Food and Drug Administration when ingested orally, these substances cause severe lung injury when heated to temperatures between 180 and 230°C and inhaled as an aerosol.

This disease outbreak is evolving rapidly. There appears to be some difference of opinion on whether e-cigarette, or vaping, product use-associated lung injury (EVALI) is best described as exogenous lipoid pneumonia or chemical pneumonitis [4]. However, the likely diagnostic path and treatment (trial and failure of albuterol and antibiotics, treatment with steroids, respiratory support and antibiotics to suppress secondary infections) remain the same. This case, with the positive oil red O staining for lipid vacuoles in macrophages, provides further evidence for the role of lipid. The patient described in this case disposed of his THC oil and vape pens after a diagnosis of EVALI was made; therefore, we were unable to collect samples for laboratory testing. Thus, this case does not inform the current debate over the role of tocopheryl acetate (vitamin E acetate) in the development of the disease.

## 3. Conclusion

The incidence of reported e-cigarette and vaping-induced lung injury has grown precipitously over the past year. Of the documented cases so far, the prevailing symptoms of THC-containing vape pens appears to be relatively consistent across many cases. While symptoms are nonspecific, including shortness of breath, asthenia, myalgias, fever, and cough, as clinicians, we must be vigilant to link this exposure and timing with lung diseases. Many patients are initially misdiagnosed and, therefore, continue using THC-containing vape pens. Our case demonstrates a common radiographic finding, diagnostic work-up, and histopathology to confirm our case for a vaping-induced lung disease, specifically exogenous lipoid pneumonia. While a definitive course of treatment remains undefined in these patients, treatment across many cases appears to show promise with conservative measures. Some patients recover with near baseline functioning in a week while others have taken months [7, 14].

The presumption that vaping is a completely safe alternative to conventional cigarette or marijuana smoking now appears to be challenged by the outbreak of EVALI. Because the patient disposed of his vape pens and cannabis extracts, we could not exclude the possibility that lung injury was caused by some contaminants. Other possible contributors to his severe adverse lung reaction include the methodology used to extract the cannabis, the heating temperature, and the diluents and flavorings. It is unclear whether or not our patient improved due to a cessation of the exposure or the medical therapy. Regardless, our patient survived with antibiotics, steroids, and supportive care. Further studies are needed to confirm our observation, to identify ingredients, and to better understand the pathophysiology of the disease.
